# Natural variations in *SlSOS1* contribute to the loss of salt tolerance during tomato domestication

**DOI:** 10.1111/pbi.13443

**Published:** 2020-07-24

**Authors:** Zhen Wang, Yechun Hong, Yumei Li, Huazhong Shi, Juanjuan Yao, Xue Liu, Fuxing Wang, Sanwen Huang, Guangtao Zhu, Jian‐Kang Zhu

**Affiliations:** ^1^ Shanghai Center for Plant Stress Biology and Center for Excellence in Molecular Plant Sciences Chinese Academy of Sciences Shanghai China; ^2^ University of Chinese Academy of Sciences Shanghai China; ^3^ The AGISCAAS‐YNNU Joint Academy of Potato Sciences Yunnan Normal University Kunming China; ^4^ Department of Chemistry and Biochemistry Texas Tech University Lubbock TX USA; ^5^ Genome Analysis Laboratory of the Ministry of Agriculture Agricultural Genomics Institute at Shenzhen Chinese Academy of Agricultural Sciences Shenzhen China; ^6^ Department of Horticulture and Landscape Architecture Purdue University West Lafayette IN USA

**Keywords:** natural variation, salt tolerance, SOS1, tomato domestication

Soil salinity is a major constraint on crop cultivability and productivity worldwide (Shabala, 2013). The ion toxicity caused by high salinity is alleviated by the adjustment of cellular Na^+^ and K^+^ homeostasis through the functions of ion transporters such as SOS1 (Salt Overly Sensitive 1) and HKT1 (High‐Affinity Potassium Transporter 1). SOS1 is a plasma membrane Na^+^/H^+^ antiporter mediating Na^+^ extrusion in root epidermal cells to reduce Na^+^ accumulation in plants and in the parenchyma cells of root and shoot xylems to promote Na^+^ translocation from root to shoot, whereas the Na^+^ transporter HKT1 mediates retrieval of Na^+^ from the xylem and may contribute to Na^+^ recirculation from shoot to root (Zhu, 2016). Natural variations in *HKT1* have been implicated in salt tolerance in several plant species (An *et al*., 2017). However, the role of natural variations in *SOS1* in adaptation to salt stress has not been reported.

Tomato (*Solanum lycopersicum*) is one of the most consumed fruit and vegetable crops in the world (Zhu *et al*., 2018). The wild ancestor of tomato is adapted to highly saline coastal habitats, while cultivated varieties have lost salt tolerance during domestication for larger fruit (Pailles *et al*., 2020). By using a genome‐wide association approach, we recently identified genetic variations in the Na^+^–K^+^ transporter *SlHAK20* responsible for the variations in root Na^+^/K^+^ ratio and the loss of salt tolerance during tomato domestication (Wang *et al*., 2020). Here, we report that genetic variations in *SlSOS1* also contribute to the phenotypic variation of salt tolerance in tomato. We collected 326 tomato accessions from the original association population, including 33 wild accessions of *S. pimpinellifolium* (PIM), 99 domesticated accessions of *S. lycopersicum var. cerasiforme* (CER) and 194 improved accessions of *S. lycopersicum* (BIG). The sequence variations in *SlSOS1* among the 326 accessions were identified based on the reference genome (Sato *et al*., 2012). Association analyses revealed two synonymous variations in the coding region and three variations in the promoter region of *SlSOS1* significantly associated with root Na^+^/K^+^ ratio (Figure 1a). The SNP‐659 variation in the promoter was found to be the most significantly associated with root Na^+^/K^+^ ratio (*P* = 1.40 × 10^−12^), and the other two adjacent variations in the promoter, SNP‐334 (G/A) and SNP‐335 (C/T), were completely in linkage disequilibrium (LD, *r* ^2^ = 1) with SNP‐659 and thus were also strongly associated with root Na^+^/K^+^ ratio (Figure 1a). Sequence analysis identified that SNP‐334 and SNP‐335 are within a known CRT/DRE *cis*‐element with the core sequence of CCGAC, and the promoters containing this *cis*‐element can be recognized and activated by the CBF/DREB transcription factors in response to stress conditions (Yamaguchi‐Shinozaki and Shinozaki, 2005). We thus classified the 326 accessions into two haplotype groups, Hap1 (haplotype group 1; *n* = 45) and Hap2 (*n* = 281), according to these two variations. Statistically, the accessions in Hap 1 showed significantly lower root Na^+^/K^+^ ratios than those in the larger group Hap2 (*P* = 1.78 × 10^−10^). Since root Na^+^/K^+^ ratio is negatively correlated with salt resistance in tomato, Hap1 and Hap2 were defined as the tolerant and sensitive alleles of *SlSOS1*, respectively (Figure 1b). The distribution of these two alleles in PIM, CER and BIG groups indicated that salt tolerance was gradually lost during tomato domestication and improvement from PIM to CER and then from CER to BIG for larger fruit (Figure 1c).

**Figure 1:**
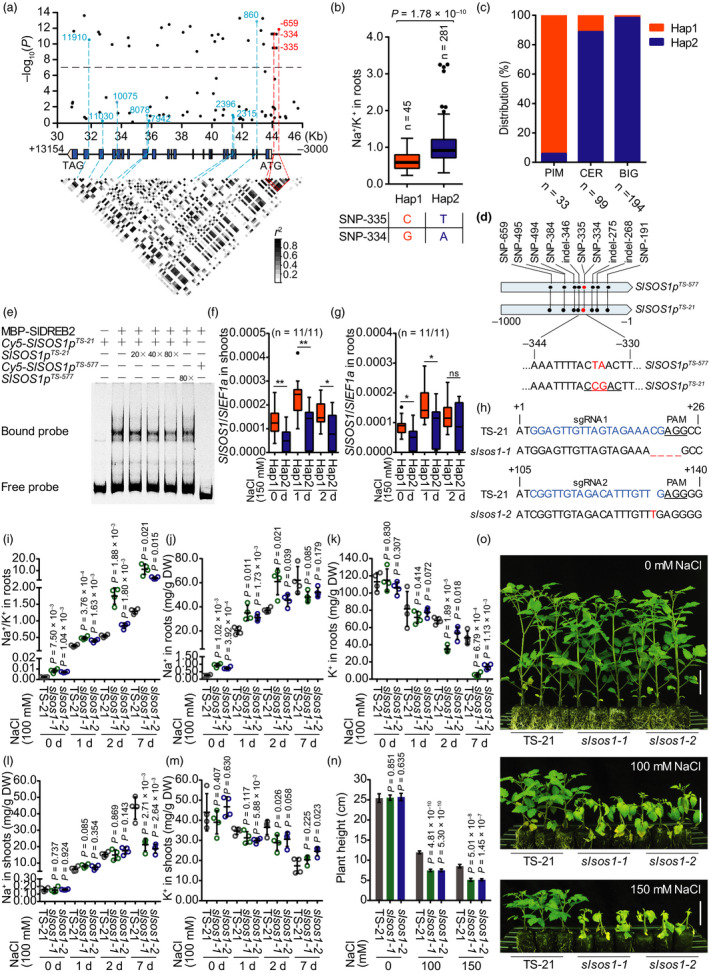
The natural variations in *SlSOS1* are associated with root Na^+^/K^+^ ratio and salt tolerance in tomato. (a) *SlSOS1*‐based association mapping and pairwise LD analysis. The variants in the promoter and coding region are highlighted in red and blue, respectively. Each variant is connected to the pairwise LD diagram with a dashed line. Red lines indicate strong LD values of SNP‐659, SNP‐335 and SNP‐334 in the promoter region. (b) Haplotypes of *SlSOS1* in the tomato population analysed based on SNP‐334 and SNP‐335. (c) The distribution of *SlSOS1* alleles in PIM, CER and BIG groups. The n indicates the number of accessions. (d) A schematic diagram showing the *SlSOS1^TS‐21^* and *SlSOS1^TS‐577^* promoter regions. The dots represent nucleotide variations. The nucleotides of SNP‐334 and −335 are indicated in red. The sequence of CRT/DRE motif in the *SlSOS1^TS‐21^* promoter is underlined. (e) Relative binding affinity of SlDREB2 to the CRT/DRE motifs in *SlSOS1* promoter. Reciprocal competitive EMSA to determine the binding of recombinant MBP‐SlDREB2 protein to the promoter region containing the CRT/DRE motifs of *SlSOS1^TS‐21^* was carried out using the indicated Cy5‐labelled probes and unlabelled competitors. The same region of *SlSOS1^TS‐577^* promoter was used for analysis. (f, g) *SlSOS1* expression in the Hap1 and Hap2 alleles in shoots (f) and roots (g) without or with salt stress treatment. Eleven accessions from each haplotype were used in this experiment. (h) Genomic sequence showing the mutations in *SlSOS1* gene generated using CRISPR/Cas9 system in the TS‐21 wild variety. The sgRNA target sites are indicated in blue. The PAM sequence is underlined. (i‐k) Na^+^/K^+^ ratio (i) and the contents of Na^+^ (j) and K^+^ (k) in roots of *slsos1‐1*, *slsos1‐2* and TS‐21 wild‐type plants. Data are shown as means ± SD (n = 4). (l, m) The contents of Na^+^ (l) and K^+^ (m) in the shoots of *slsos1‐1*, *slsos1‐2* and TS‐21 wild‐type plants. Data are shown as means ± SD (n = 4). (n, o) Salt tolerance of *slsos1* mutants and wild type (TS‐21) indicated by plant height (n) and growth phenotype (o). Three‐week‐old *slsos1‐1*, *slsos1‐2* and TS‐21 plants were treated with 0, 100 or 150 mM NaCl for 1 week, followed by recovery for 1 week, and then, plant height was measured. Values are means ± SD (n = 6 plants of each genotype). Bars, 5 cm. In the box plots of (b), (f) and (g), boxes indicate the range of the percentiles of the total data using Turkey method, centre values are medians, dashed lines indicate variability outside the upper and lower quartiles, and dots denote outliers. n denotes the number of accessions belonging to each haplotype group. Statistical significance was determined by Student’s t‐test (*P < 0.05, **P < 0.01).

We chose *SlSOS1^TS‐21^* in Hap1 and *SlSOS1^TS‐577^* in Hap2 as representatives of these two groups for further study. The variant promoter *SlSOS1p^TS‐21^* with C and G at the position of SNP‐335 and SNP‐334 maintained the CRT/DRE core *cis*‐element, whereas the *SlSOS1p^TS‐577^* with T and A at the SNP‐335 and SNP‐334 disrupted the core *cis*‐element (Figure 1d). To assess whether the variations in this *cis*‐element contribute to *SlSOS1* expression, we analysed the binding capacity of the CRT/DRE variants with SlDREB2, a known salt‐inducible DREB transcription factor in tomato recognizing CRT/DRE motif and inducing the expression of target genes (Hichri *et al*., 2016). Reciprocal competitive electrophoretic mobility shift assay (EMSA) showed strong and specific binding of SlDREB2 to the CRT/DRE motif in the promoter region of *SlSOS1^TS‐21^*, whereas no binding was observed in the region of *SlSOS1^TS‐577^* promoter with disrupted CRT/DRE motif (Figure 1e). Gene expression analysis revealed that the transcript levels of *SlSOS1* are increased in the Hap1 varieties in response to high salinity, and this up‐regulation was markedly lower in the Hap2 accessions (Figure 1f and g).

The critical role of *SlSOS1* in salt tolerance was further validated by analysing the knockout mutants of tomato (Figure 1h). We generated two mutant alleles, *slsos1‐1* and *slsos1‐2*, and analysis of root Na^+^/K^+^ ratio showed a significantly higher Na^+^/K^+^ ratio in the mutants than in the wild‐type TS‐21 (Figure 1i). After 1–2‐day salt treatment, the mutants accumulated more Na^+^ in roots but similar levels of Na^+^ in shoots when compared with wild‐type plants, whereas after 7 days of treatment, the mutants showed a similar level of Na^+^ in roots but accumulated markedly lower Na^+^ in shoots compared with the wild type (Figure 1j and l). A sharp reduction in K^+^ content in the mutants was only observed in the roots after salt treatment for 2 and 7 days (Figure 1k and m). These results suggest that SlSOS1 controls Na^+^ and K^+^ homeostasis in tomato roots and shoots under salt stress conditions. Furthermore, phenotype analysis showed that *slsos1‐1* and *slsos1‐2* mutants were clearly more sensitive to salt stress than wild type plants (Figure 1n and o), which indicates that, like the *Arabidopsis SOS1* (Shi *et al*., 2003), *SlSOS1* also plays a crucial role in salt tolerance in tomato. Overall, our findings indicate that natural variations in the promoter of *SlSOS1* disrupting the SlDREB2‐binding *cis*‐element result in reduced expression of *SlSOS1* and increased salt sensitivity in the cultivated tomato due to selection during domestication. The wild *SlSOS1* variants provide valuable natural resources and genetic markers for improvement in salt tolerance in tomato.

## Author contributions

S.W.H. and J.‐K.Z. conceived the project. Z.W., Y.H. and G.Z. designed the research. Z.W., Y.H., J.Y., X.L. and F.W. performed most of the biological experiments. G.Z. and Y.L. carried out the association mapping and pairwise LD analysis. Z.W. performed tomato transformation. Z.W., G.Z. and H.Z.S. performed the data analysis. Z.W. and H.Z.S. drafted the manuscript. G.Z. and J.‐K.Z. revised the manuscript.

## Conflict of interest

The authors declare no competing interests.
